# Silver Meshes for Record-Performance Transparent Electromagnetic Interference Shielding

**DOI:** 10.1021/acsami.3c02088

**Published:** 2023-06-14

**Authors:** Mingxuan Li, Mehdi Zarei, Khashayar Mohammadi, S. Brett Walker, Melbs LeMieux, Paul W Leu

**Affiliations:** † Department of Chemical Engineering, University of Pittsburgh, Pittsburgh, Pennsylvania 15261, United States; ‡ Department of Mechanical Engineering, University of Pittsburgh, Pittsburgh, Pennsylvania 15261, United States; ¶ Department of Civil Engineering, University of Waterloo, Waterloo, Ontario N2L3G1, Canada; § Electroninks Incorporated, Austin, Texas 78744, United States; ∥ Department of Industrial Engineering, University of Pittsburgh, Pittsburgh, Pennsylvania 15261, United States

**Keywords:** EMI shielding, Metal ink, Reactive ion etching, Photolithography, Metal mesh, Transparent electrode

## Abstract

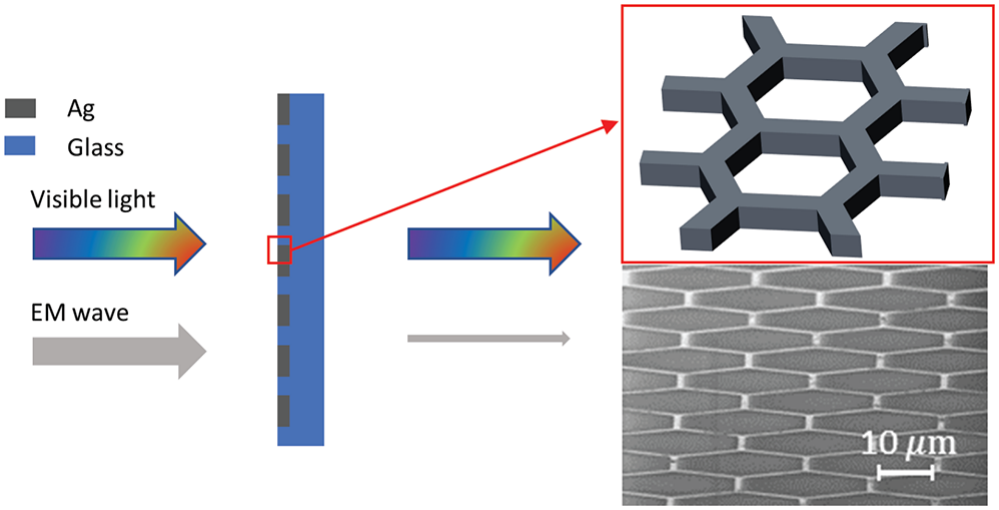

We present a simulation and experimental study of silver meshes to determine their performance for transparent electromagnetic interference (EMI) shielding. Simulations were employed to study the effects of the silver mesh’s width, pitch, and thickness on EMI shielding efficiency (SE) in the 8–18 GHz frequency range and transparency in the visible spectrum. We demonstrate a scalable, facile fabrication method that involves embedding meshes in glass by etching trenches in glass and filling and curing reactive particle-free silver ink in these trenches. Our silver meshes achieve 58.4 dB EMI SE with 83% visible light transmission and 48.3 dB EMI SE with 90.3% visible transmission. The combination of high-conductivity silver, small widths (1.3 to 5 μm), and large thicknesses (0.5 to 2.0 μm) enables the best performance of metal meshes as well as single-sided shielding materials for transparent EMI shielding, as reported in the literature.

## Introduction

The increasing complexity and widespread use of electronic devices and systems has led to growing demand for new types of electromagnetic interference (EMI) shielding materials and fabrication methods.^1^ EMI shielding materials are needed to reduce the impact of surrounding radiation on electronic components and to prevent their functionality or accuracy from being compromised or their lifetimes from being shortened.^2,3^ In addition to protecting electronic components, EMI shielding can provide security from sensitive information being compromised^2,4^ or from electronic systems being disabled by electromagnetic pulse weapons or attacks.^5,6^ EM radiation may also have negative effects on human health.^4^

In many optoelectronic devices, such as heated windows, smart displays, and wearable electronics, it is required that the shielding material achieves both high shielding efficiency (SE) and high visible light transmission (*T*). Transparent EMI shielding has, thus, been the focus of much research. One category of transparent EMI shielding consists of thin film structures. High conductivity metals such as silver (Ag), copper (Cu), and nickel (Ni) are used to form a conductive film to reflect and absorb EM waves. Materials with a high refraction index such as indium tin oxide (ITO) or zinc oxide (ZnO) may be used to surround the metal film in order to increase transparency by leveraging destructive interference to reduce reflected light.^7^ It has been reported that ITO/Ag–Cu/ITO film structures have achieved 26 dB SE and 96.5% visible transmission.^8^ ZnO/Ag/ZnO sandwich structures have been reported with 35 dB SE and 88.9% transmission in the visible range.^9^ The theoretical performance limits of these thin film structures were explored in our previous paper.^7^ We determined that titania/Ag/titania (TiO_2_/Ag/TiO_2_) film structures could achieve 41.2 dB SE with 90.8% visible transmission. These simulations suggest that it is unlikely for these sandwich structures to achieve higher than 45 dB SE while maintaining over 90% visible transmission.

Another category of transparent EMI shielding consists of metal meshes.^10,11^ Since visible light is comprised of EM radiation with smaller wavelengths than microwaves, this approach uses metal meshes with an intermediate pitch to enable visible light to pass through the open areas, while blocking the larger wavelength microwaves. Ni meshes on ITO have achieved 92% optical transmission with 40 dB SE.^12^ The meshes were fabricated by patterning photoresist through laser direct-writing followed by nickel electrodeposition to create meshes with widths of 5 μm and thicknesses ranging from 2.5 to 6.0 μm.^12^ This approach was developed further to achieve double-layer nickel meshes on PET to achieve an average SE of 42.5 dB and a transmission of 88.7%.^13^ The width of the Ni mesh is 6.5 μm and thickness is 4 μm. Multi-ring Al meshes have exhibited 90% transmission with 27 dB SE.^14^ These multi-ring meshes were fabricated by photolithography followed by dry etching of the aluminum and achieved line widths of about 2.5 μm and thickness of 0.2 μm. Cu meshes with an average SE of 41 dB and transmission of 85% have been fabricated by thermal evaporation of Cr/Cu onto a crack lithography pattern, which created meshes with a width of 5 μm and thickness of up to about 1 μm.^15^ Liang et al. have demonstrated Cu meshes have been fabricated by ion beam etching Cr/Cu films after photoresist patterning to achieve 45 dB SE and 85% transmission.^16^ These Cr/Cu meshes have a width of 0.85 μm and thickness of 0.5 μm. These Cr/Cu meshes represent the highest performing transparent EMI shielding for meshes with visible transparency greater than or equal to 80% transmission with a SE of 45 dB. The best performing EMI shielding for meshes with visible transparency greater than or equal to 90% are the Ni meshes by Jiang et al. with widths of 5 μm and thicknesses ranging from 2.5 to 6.0 μm that have demonstrated an SE of 40 dB.^12^

The EMI SE performance of metal nanomeshes, such as those of Ni, Al, and Cu, may, in principle, be improved with Ag, which has the highest conductivity of all metals. However, results with Ag meshes have been inferior to those of other metals. Ag metal meshes have been reported to achieve 90.5% optical transmission with 26 dB SE.^17^ These Ag metal meshes were fabricated by electric-field-driven microscale 3D printing and have a line width of 26 μm. Ag meshes patterned by photolithography and metal lift-off have exhibited a transmittance of 92% and SE of 28.8 dB.^18^ These meshes have a width of 6 μm and a thickness of 0.2 μm. Ag/Cu and Ag/Ni meshes have also been fabricated by sputtering Ag onto a crack lithography template followed by galvanic deposition of Cu or Ni.^19^ An average SE of 38.5 dB with transmission of 85.4% was achieved with a width of 5.5 μm and a thickness of about 0.25 μm. While Ag has the potential for achieving a higher performance in transparent EMI shielding compared to other metals due to its high conductivity,^20^ this improved performance has yet to be realized.

In this paper, we present both a simulation and an experimental study of metal meshes for transparent EMI shielding. We performed simulations to provide for understanding of the relationships between width, pitch, and thickness of metal meshes and their influence on EMI SE and visible transparency. Our simulations indicate that a small width and large thickness are key to achieving high performance. These simulations suggest that the large widths and small thicknesses of Ag meshes fabricated thus far are the reasons for their suboptimal performance.

We demonstrate the facile, scalable fabrication of Ag meshes with an unprecedented combination of small widths and large thicknesses to achieve record performance. Previous transparent EMI shielding work with metal meshes has not been demonstrated with reactive ink-based fabrication approaches. Glass-embedded metal meshes were fabricated by etching trenches into glass using lithography and reactive ion etching (RIE). Reactive, high conductivity Ag ink was used to fill the trenches via a low temperature curing process (under 110 °C). We demonstrate Ag meshes that exhibit an EMI SE of 58.4 dB with 83% visible transmission and Ag meshes with an EMI SE of 48.3 dB and 90.3% visible transmission. This is the record highest transparent EMI shielding performance for metal meshes and the highest performance for single-sided structures of any kind. The metal meshes have a width of 1.6 μm and thickness of 0.8 μm and a width of 3.5 μm and thickness of 1.0 μm, respectively. Metal meshes embedded in glass substrates with thicknesses up to 2.0 μm, which are planarized with the glass surface, are demonstrated and may be utilized for a variety of optoelectronic applications. These metal meshes achieve the best transparent EMI shielding performance due to the combination of the use of high-conductivity silver, small widths, and large thicknesses.

## Results and Discussion

**Figure 1:**
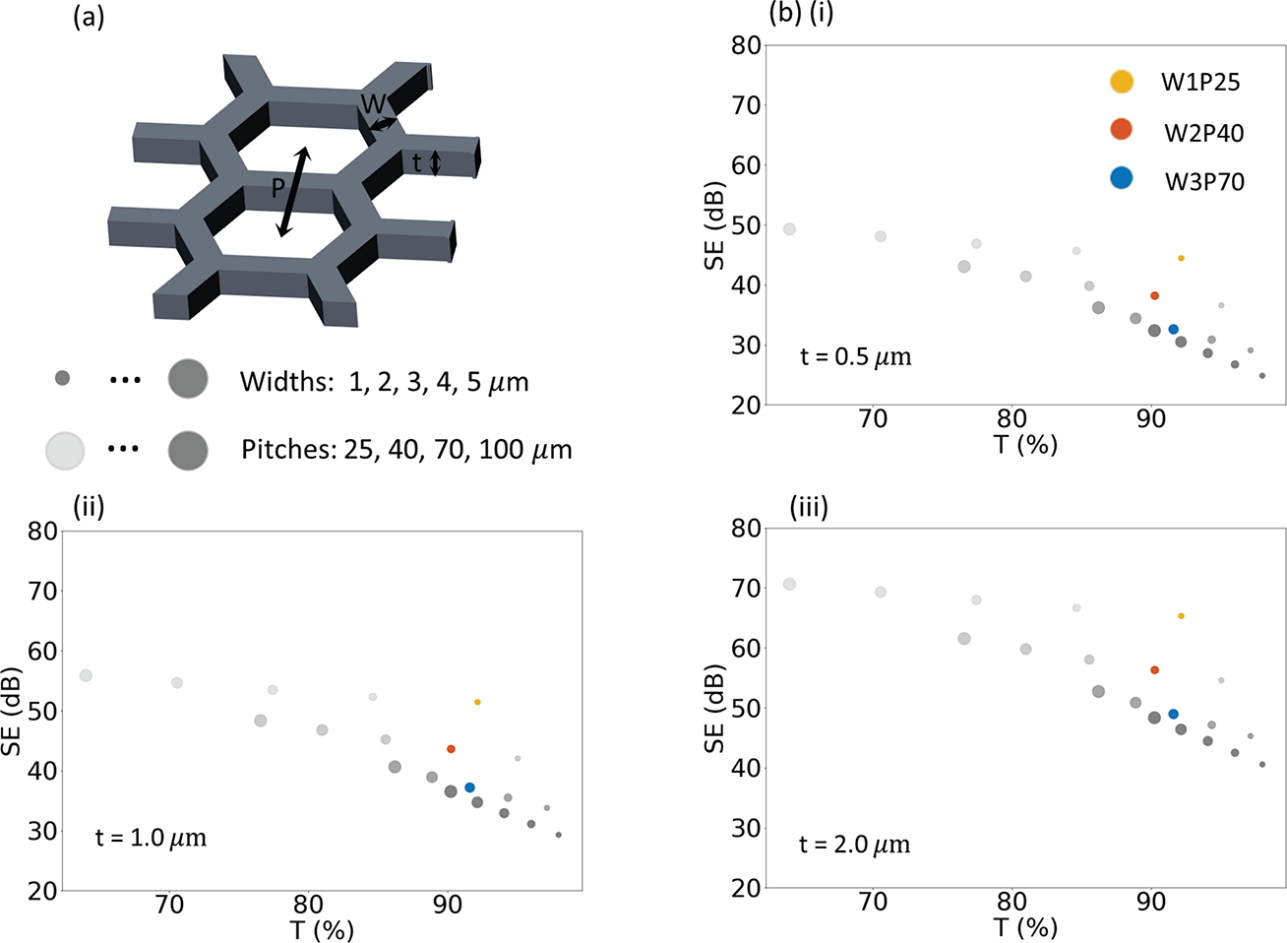
High-frequency electromagnetic field simulation results of average EMI shielding efficiency at 8–18 GHz for Ag meshes with different pitches, widths, and thicknesses. (a) Schematic of hexagonal Ag mesh defined by width *W*, pitch *P*, and thickness *t*. (b) Simulation results for metal meshes with widths of 1, 2, 3, 4, 5 μm, pitches of 25, 40, 70, 100 μm, and for three thicknesses of (i) 0.5, (ii) 1, and (iii) 2 μm.

Figure 1 shows (a) a schematic of the metal mesh and (b) the simulation results for metal meshes. The metal meshes are defined by their width *W*, pitch *P*, and thickness *t*. High-frequency electromagnetic field simulation using the finite element method was used to predict EMI SE performance.^21−23^ The details of the simulations are provided in Table S1. Since the pitches studied are much larger than that of visible light, the 550 nm transmission was calculated from the geometric ratio of open area to total area:
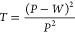
1The simulation results are shown in Figure 1b for three different thicknesses: *t* = (i) 0.5, (ii) 1.0, and (iii) 2.0 μm. The conductivity of the silver is assumed to be 1.3 × 10^7^ S/m or 20% that of bulk silver. The size of the circle in the plots is proportional to the width of the meshes, where the widths of 1, 2, 3, 4, and 5 μm were simulated. The darkness of the circle indicates the pitch of the meshes, and four different pitches of 25, 40, 70, and 100 μm were simulated.

The trade-offs between EMI SE and optical transparency can be observed in the simulation results. Increasing the width for the same thickness and pitch results in higher EMI SE due to increased metal coverage at the expense of visible transparency. Conversely, as the pitch increases while keeping the same thickness and width reduces EMI SE, while improving visible transparency. The two metrics of EMI SE and optical transparency can be evaluated through the concept of Pareto optimality, where it is impossible to improve one metric without making the other aspect worse. In this context, the simulation results indicate that smaller width meshes provide better results compared to meshes with larger width. For the same transparency, a higher EMI SE may be achieved with smaller width meshes. Furthermore, for the same width and pitch mesh, it is desirable to fabricate thicker meshes, which can improve the SE for a given *T*. As the pitch of meshes is easily adjustable, the key to obtaining high-performance EMI SE is fabricating meshes of small width and large thickness. Increasing thickness is crucial for enhancing performance because a 1 μm increase in thickness has a greater impact on transparent EMI SE than a 1 μm decrease in width.

The smallest width Ag meshes that have been fabricated thus far are Ag/Cu meshes with 5.5 μm width.^19^ These meshes have a thickness of only 0.25 μm. This large width and small thickness limit the performance of these meshes to an EMI SE of 38.5 dB and visible transmission of 85.4%. There is a need to fabricate silver meshes with smaller widths and larger thicknesses to enable better performance. Toward this goal, we demonstrate a large-area, low-cost method to fabricate Ag meshes with small width and large thickness. Three meshes of varying nominal widths *W* of 1, 2, and 3 μm and pitches of 25, 40, and 70 μm, respectively, were studied in more detail through experiments. The three meshes are labeled W1P25, W2P40, and W3P70, which indicate their width and pitch in microns. For example, W1P25 refers to a mesh width of 1 μm and pitch of 25 μm. These three meshes are shown in Figure 1 with yellow, red, and blue circles, respectively.

**Figure 2:**
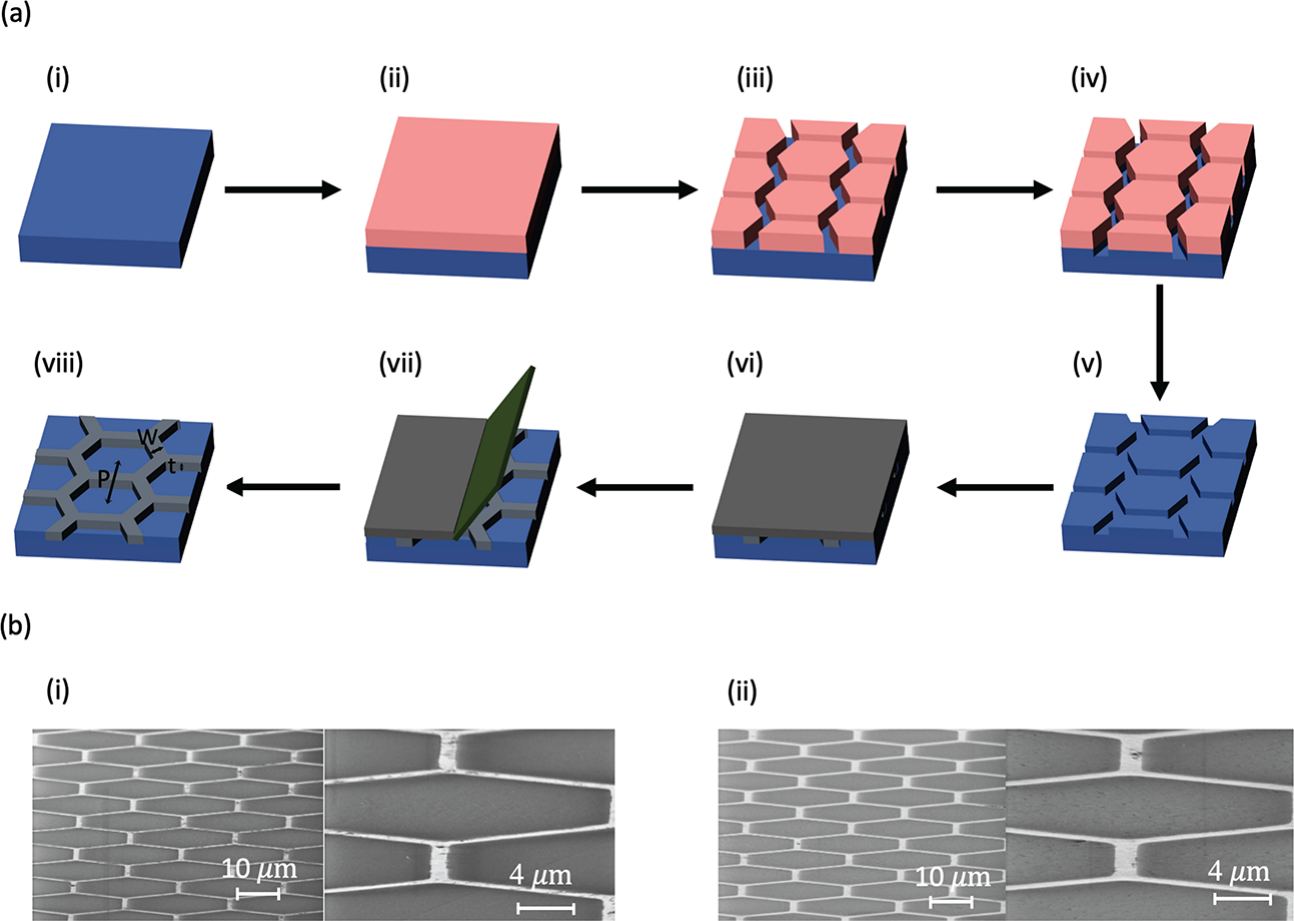
(a) Schematic of glass-embedded Ag meshes fabrication process: (i) transparent glass substrate, (ii) photoresist coating, (iii) photolithography, (iv) reactive-ion etching, (v) photoresist removal, (vi) Ag ink coating and curing, (vii) removal of Ag by a razor blade, and finally (viii) glass-embedded Ag mesh. The Ag meshes are hexagonal arrays embedded into a glass substrate, defined by width *W*, pitch *P*, and thickness *t*. (b) Scanning electron microscopy (SEM) images of (i) W1P25t0.5 and (ii) W1P25t0.8 samples taken at an 80° angle from directly overhead with actual widths of 1.3 and 1.6 μm, respectively.

Figure 2a shows a schematic of the fabrication process. A glass substrate (Figure 2a,i) is coated with photoresist (Figure 2a,ii), and a hexagonal pattern is transferred to this photoresist using maskless photolithography (Figure 2a,iii). The hexagonal pattern is transferred into the glass substrate by RIE (Figure 2a,iv). Then the photoresist is stripped (Figure 2a,v). Ag ink is coated onto the glass by spin coating and then cured (Figure 2a,vi). The Ag is scratched off using a blade (Figure 2a,vii) revealing the hexagonal structure (Figure 2a,viii). The coating, curing, and scratching steps are repeated again to ensure complete trench filling.

Six different metal mesh samples were fabricated of varying widths, pitches, and thicknesses. For each of three different mesh pitch and width combinations (W1P25, W2P40, and W3P70), two different thickness meshes were fabricated. Figure S1 shows the trench profile of the six different samples as characterized by optical profilometry prior to the Ag ink coating. A consistent vertical etch rate of ∼1.2 nm/s is observed in the samples. However, while the etching is primarily vertical, there is also some lateral etching, resulting in the etched trenches have a trapezoidal cross-section as opposed to a rectangular one. The Ag ink coating and scratching are performed twice to ensure complete trench filling. Figure S2 shows optical profilometry analysis of one of the metal mesh samples (W3P70t1.0) prior to the Ag ink coating, after one coat, and then after a second coat. The cured Ag only partially fills the trenches due to the large reduction in volume from solvent evaporation during the Ag ink curing process. The final cured silver is planar with the unetched glass, which may be beneficial for applications such as organic light emitting diodes (OLEDs) and solar cells. The fabricated samples are uniform with no traces of silver residue in between the meshes. Atomic force microscopy (AFM) characterization of the final metal mesh reveals very small roughness. Figure S3 illustrates the characterization of the surface roughness of the same metal mesh sample by AFM. The root-mean-square roughness (*R*_q_) of the silver is 6.7 nm.

The width and thickness of the samples that were fabricated were measured by scanning electron microscopy (SEM) and the samples are summarized in Table 1. Figure 2b shows SEM images of the W1P25t0.5 and W1P25t0.8 samples. These samples were etched for 400 and 700 s, respectively. The W1P25t0.5 sample has a measured width of 1.3 μm and a thickness of 0.5 μm, while the W1P25t0.8 sample exhibits a measured width of 1.6 μm and a thickness of 0.8 μm.

**Table 1:** Summary of the Etch Time, Measured Thickness, and Measured Width for Various Samples Fabricated

Number	Sample	.Etch Time (s)	*t* (μm)	*W*_m_ (μm)
1	W1P25	400	0.5	1.3
2	W1P25	700	0.8	1.6
3	W2P40	800	1.0	2.8
4	W2P40	1300	1.6	3.2
5	W3P70	800	1.0	3.5
6	W3P70	1600	2.0	5.0

Based on the simulation results discussed, it is desirable to fabricate metal meshes with a width *W* as narrow as possible and a thickness *t* as large as possible. The pitch can be set arbitrarily. However, the meshes width and pitch are not independent parameters. In order to achieve small width meshes, thinner photoresist must be used. The thicknesses of the photoresist for the W1, W2, and W3 samples are 0.6, 2, and 2.8 μm, respectively. This limits the thickness achievable in the metal meshes as the thickness of the photoresist limits how long the glass can be etched. There are thus trade-offs between the narrowness of width achievable and the depth of the trench achievable. This trade-off between width and thickness translates to trade-offs in transmission and SE that are achievable.

**Figure 3:**
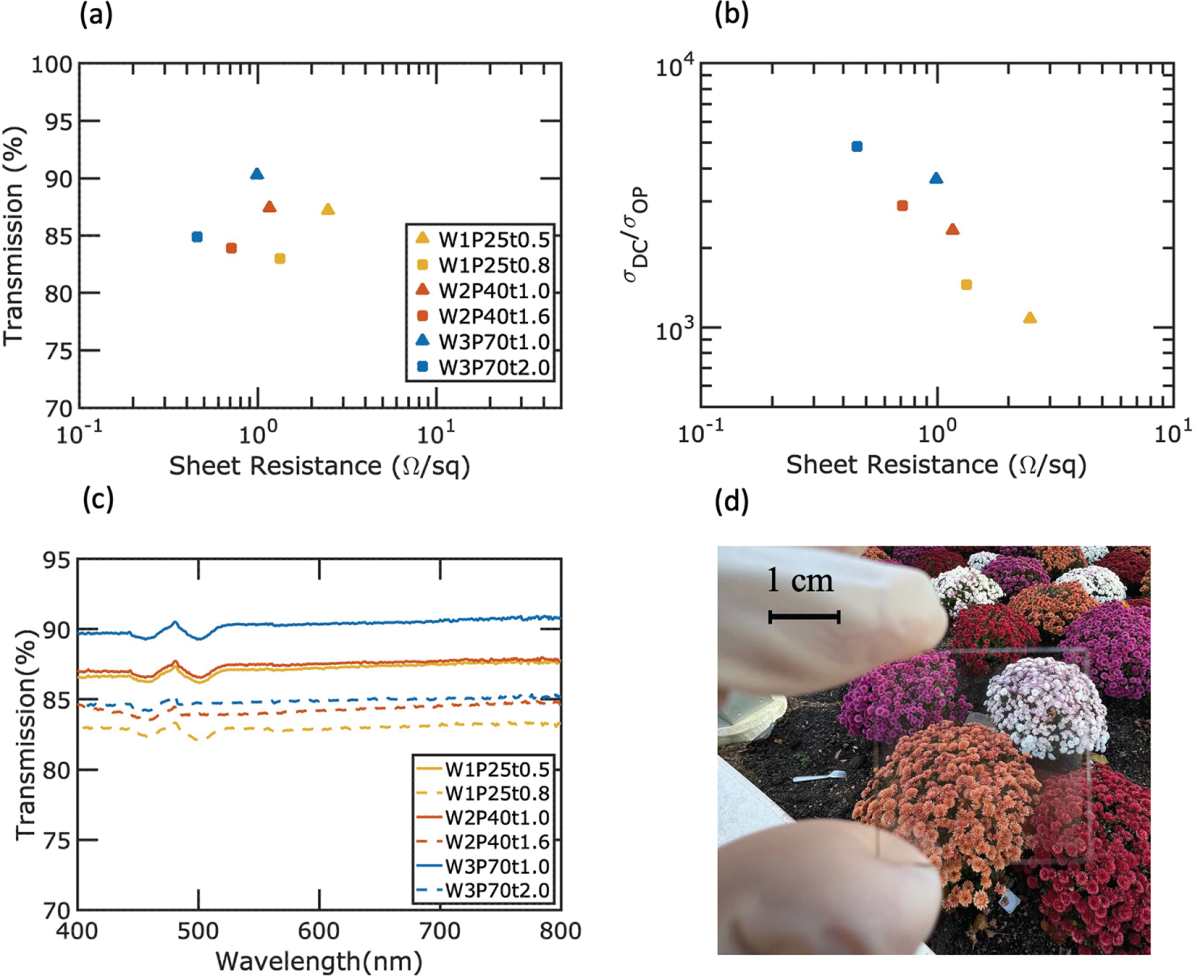
Optical performance of glass-embedded Ag meshes for three different combinations of pitch and width and different etch times: (a) transmission versus sheet resistance, (b) figure of merit σ_DC_/σ_OP_, (c) transmission versus wavelength, and (d) optical image of W1P25t0.5 with *T* = 87.2% at 550 nm.

The optical performance of the six different Ag mesh samples was characterized. Figure 3 shows the optical transmission results. Figure 3a shows the transmission at a 550 nm wavelength and the sheet resistance of the six samples. For a particular mesh width and pitch combination, the shorter etch time results in a smaller measured width and a smaller thickness (indicated with a triangle), while the longer etch time results in a larger measured width and a larger thickness (indicated with a square). Etching the samples longer thus results in a smaller transmission with a smaller sheet resistance. The transmission for all the samples is in the range of 83.0–90.3% with the sheet resistance *R*_*s*_ in the range of 0.46–2.47 Ω per sq. The direct transmission of all six samples was characterized and the hazes for all the samples are shown in Table S2. The optical transmission is primarily due to direct transmission as the haze values are all 7.0% or lower.

Figure 3b plots the figure of merit (σ_DC_/σ_OP_) for various metal meshes as a function of *R*_s_. The figure of merit is used for comparing transparent electrodes and is determined by the ratio between the dc conductivity (σ_DC_) and the optical conductivity (σ_OP_). A higher figure of merit indicates a more efficient device, which is able to transmit a larger amount of electrical current while still maintaining good optical transparency. The figure of merit is related to *T* and *R*_s_ by
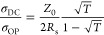
2where *Z*_0_ = 377 Ω is the free space impedance. Mesh samples achieve σ_DC_/σ_OP_ in the range of 1000–4900, which are comparable to the best transparent electrodes in the literature.^24−28^Figure 3c shows the variation of transmission versus wavelength in the visible range. The transparency spectrum across the visible wavelength range is fairly flat. Figure 3d shows the optical image of the 30 mm × 30 mm W1P25t0.5 sample, which exhibits *T* = 87.2% at a wavelength of 550 nm. The uniformity and clarity of the sample can be seen in the image.

**Figure 4:**
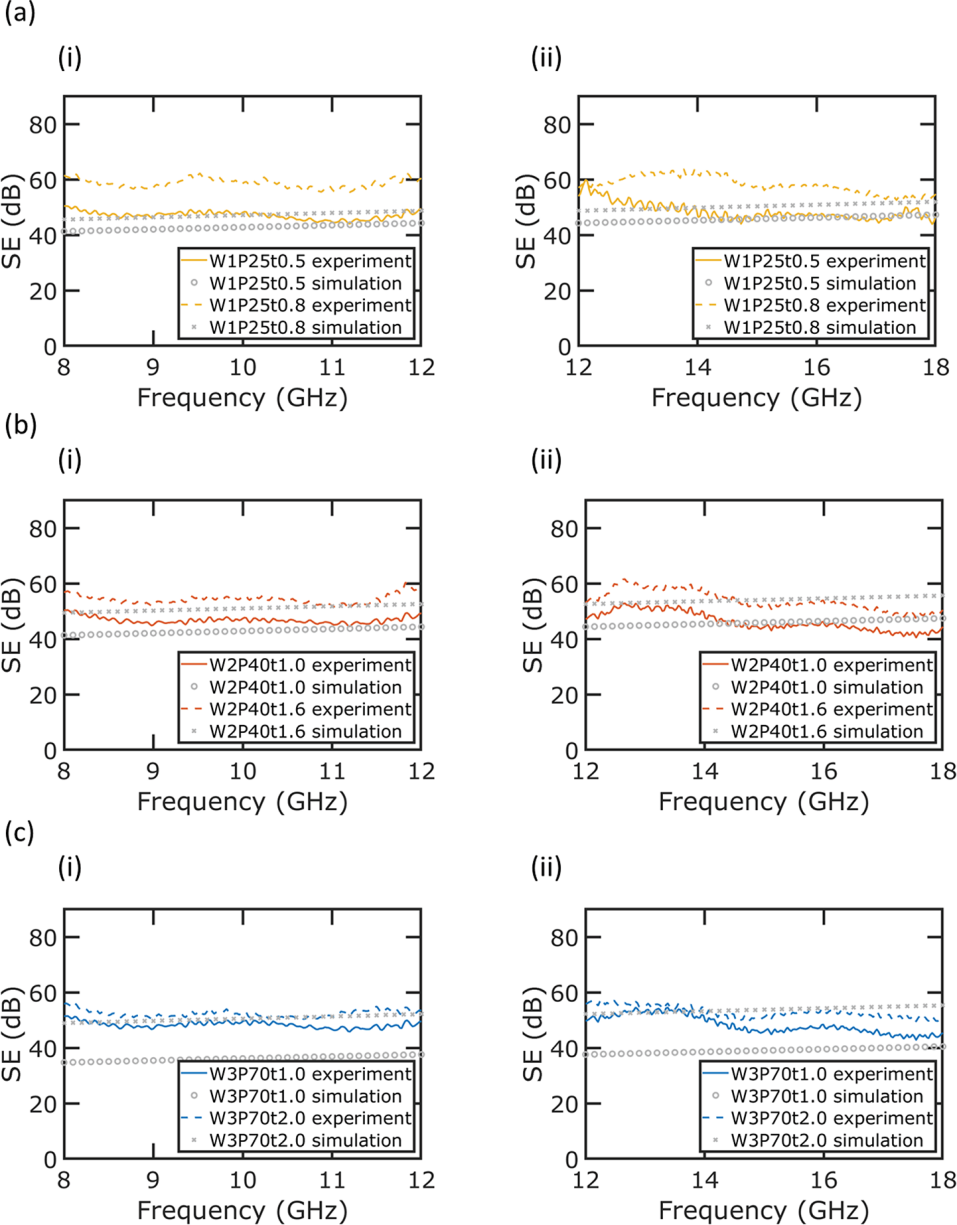
EMI shielding efficiency experimental and simulation results of (a) W1P25t0.5 and W1P25t0.8 samples, (b) W2P40t1.0 and W2P40t1.6 samples, and (c) W3P70t1.0 and W3P70t2.0 samples at (i) 8–12 GHz and (ii) 12–18 GHz.

Figure 4 shows the EMI SE for the six different metal mesh samples. EMI shielding performance was characterized by the coaxial transmission line method.^29^ The shielding effectiveness is given by
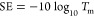
3where *T*_m_ is the transmission coefficient of the sample at the microwave frequency of interest. Figures 4a shows the EMI SE performance of the (i) W1P25t0.5 and (ii) W1P25t0.8 samples. The shorter etched sample (W1P25t0.5) has an average EMI SE of 47.1 dB at frequencies ranging from 8–12 GHz and 48.6 dB from 12–18 GHz, while the longer etched sample (W1P25t0.8) has an average EMI SE of 58.6 dB between 8–12 GHz and 58.2 dB between 12–18 GHz. Figure 4b shows the EMI SE performance of the (i) W2P40t1.0 and (ii) W2P40t1.6 samples. The shorter etched sample (W2P40t1.0) has an average EMI SE of 46.8 dB between 8–12 GHz and 45.9 dB from 12–18 GHz and the longer etched sample (W2P40t1.6) has an average EMI SE of 54.4 dB between 8–12 GHz and 53.8 dB from 12–18 GHz. The SE of the fabricated samples in this paper has a low dependency on frequency range from 8–18 GHz, which makes them suitable for a variety of applications. Figure 4c shows the EMI SE performance of (i) W3P70t1.0 and (ii) W3P70t2.0 samples. The shorter etched sample (W3P70t1.0) has an average EMI SE of 48.4 dB from 8–12 GHz and 48.3 dB from 12–18 GHz, while the longer etched sample (W3P70t2.0) has an average EMI SE of 52.5 dB from 8–12 GHz and 53.0 dB from 12–18 GHz. Simulation results for these different metal meshes are also included.

For a particular pitch and width combination, the longer etch treatment leads to a lower optical transmission and higher EMI SE. This is because the longer etch time results in a larger width due to lateral etching. Shorter etched metal meshes could be used for applications such as shielding windows and display devices,^30^ which require higher optical transmission, while longer etched meshes could be used for medical devices and military applications,^31^ which require higher EMI SE.

Table 2 provides a comparison of the simulation and experimental measurement for *T*, SE, and *R*_s_ of all the fabricated samples in this paper. The simulated *T* simply comes from eq 1, and thus only depends on the width and pitch. Simulated SE and *R*_s_ consider the measured thickness and width, which are calculated from SEM images. A silver conductivity of 1.3 × 10^7^ S/m or 20% that of bulk silver is used as described above. Good agreement can be seen between the simulation and experimental results. The experimentally measured SE tends to be higher than that of simulations. This may be because the cured Ag meshes are porous, which may result in multiple reflections and an increase in EMI SE performance due to increased absorption.

**Table 2:** Comparison of the Simulation and Experimental Results for Our Six Fabricated Metal Meshes^a^

		Simulations			Experiments		
Number	Sample	*T* (%)	SE (dB)	*R*_s_ (Ω/*sq*)	*T* (%)	SE (dB)	*R*_s_ (Ω/*sq*)
1	W1P25t0.5	89.9	44.8	1.84	87.2	48.0	2.47
2	W1P25t0.8	87.6	49.3	0.95	83.0	58.4	1.33
3	W2P40t1.0	86.5	44.9	0.58	87.4	46.3	1.16
4	W2P40t1.6	84.6	53.1	0.35	83.9	54.0	0.71
5	W3P70t1.0	90.3	38.1	0.75	90.3	48.3	0.99
6	W3P70t2.0	86.2	52.7	0.29	84.9	52.8	0.46

a The transparency (*T*) at 550 nm, average shielding efficiency (SE) from 8 to 18 GHz, and sheet resistance (*R*_s_) are all presented.

**Figure 5:**
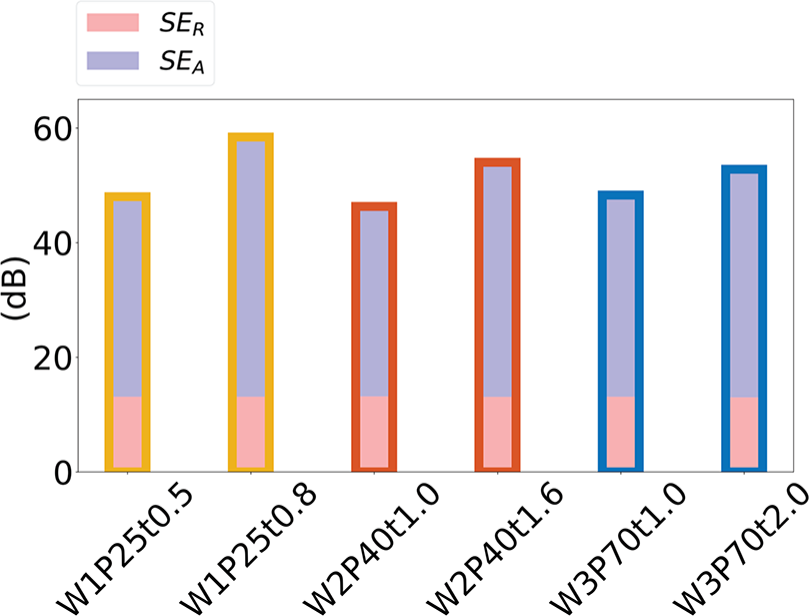
SE contribution of SE_A_ and SE_R_ for six samples.

Silver meshes exhibit a combination of reflection and absorption shielding mechanisms. Figure 5 shows the shielding contribution in the six samples from these two components: SE = SE_R_ + SE_A_, where SE_R_ represents the shielding efficiency between 8 and 18 GHz due to reflection, and SE_A_ corresponds to the shielding efficiency between 8 and 18 GHz resulting from absorption. These two components are defined by
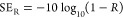
4where *R* denotes the reflection coefficient and
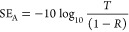
5The SE_R_ values for the six samples are all about the same at 13.1, 13.1, 13.0, 13.1, 13.2, and 13.1 dB, respectively. In contrast, the SE_A_ values for the six samples are 34.9, 45.3, 33.3, 40.9, 35.1, and 39.7 dB, respectively. Absorption is the primary mechanism of EMI shielding effectiveness for the Ag metal mesh.

**Figure 6:**
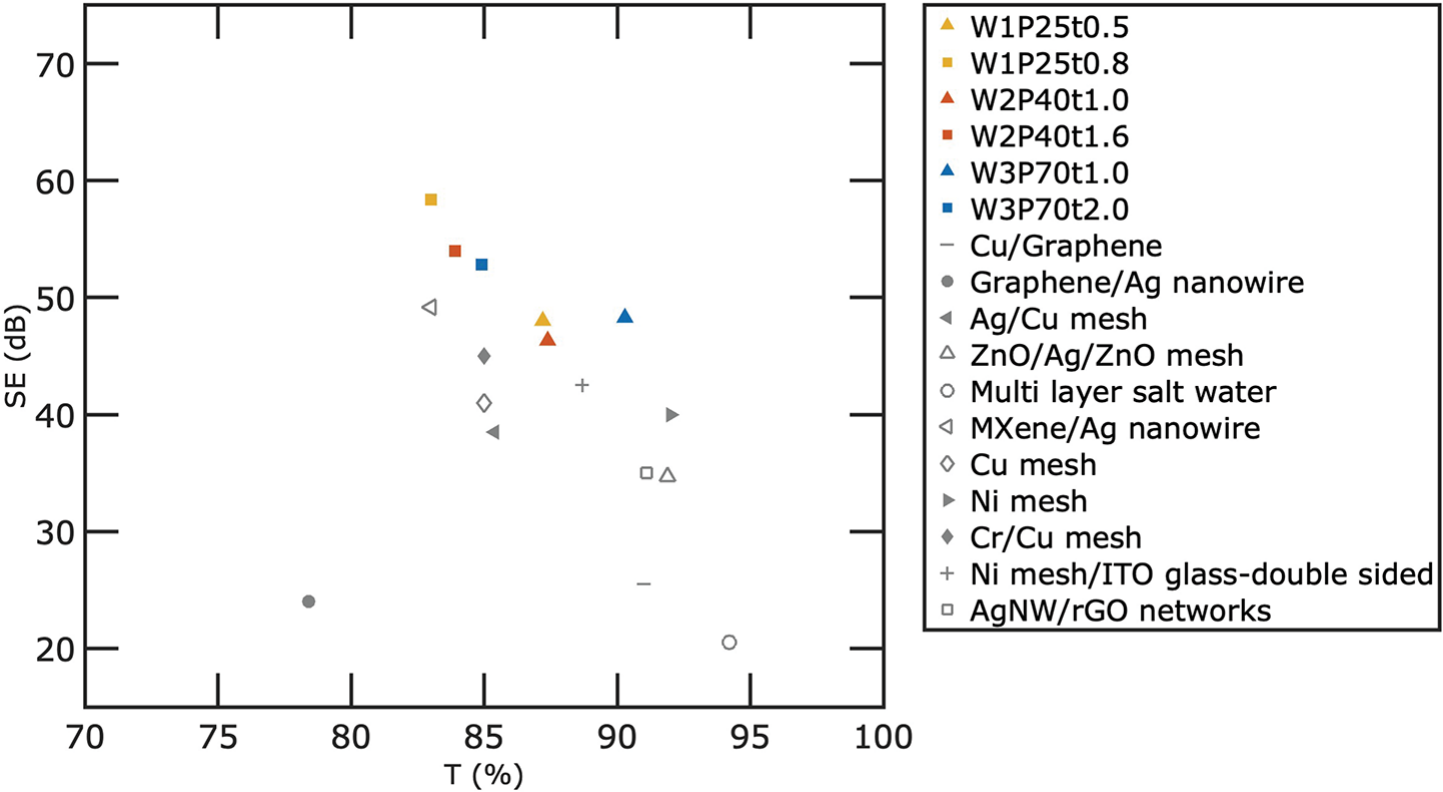
Comparison of the EMI SE (dB) and transmission (at 550 nm) of our six microgrids with other metal meshes and materials in the literature: Cu/Graphene,^32^ Ni mesh/ITO glass,^13^ Ag/Cu mesh,^19^ ZnO/Ag/ZnO,^9^ multilayer salt water,^34^ ITO/Ag–Cu/ITO,^8^ MXene/Ag nanowire,^35^ Cu mesh,^15^ Ni mesh,^12^ Cr/Cu mesh,^16^ Ni mesh-double-sided,^13^ and AgNW/rGO networks.^36^

In Figure 6, we compared EMI SE (dB) and transmission of our six fabricated metal–metal meshes with transparent EMI shielding in the literature. This includes Cu/Graphene,^32^ Graphene/Ag nanowire,^33^ Ni mesh/ITO glass,^13^ Ag/Cu mesh,^19^ ZnO/Ag/ZnO,^9^ multilayer salt water,^34^ ITO/Ag–Cu/ITO,^8^ MXene/Ag nanowire,^35^ Cu mesh,^15^ Ni mesh,^12^ Cr/Cu mesh,^16^ Ni mesh-double-sided,^13^ and AgNW/rGO networks.^36^ It should be noted that different papers investigate various frequency ranges and report SE values as maximum, minimum, or average. However, to ensure consistency with the current paper, the average SE in the range of 8–18 GHz is reported here. The details of the comparison results of this paper with other works in the literature can be found in Table S3 of the Supporting Information.

The Pareto frontier of our Ag mesh dominates that of other previous results for several reasons. First, our fabricated meshes utilize Ag, which has the highest conductivity of all the elements and is significantly higher than metals used in other metal meshes such as Cu and Ni. Second, our fabrication process enables small width and large thickness metal meshes. Our Ag meshes, with thicknesses of up to ∼2 μm, outperformed Cu meshes with lower conductivity and thickness.^16^ Ag/Cu meshes with a width of 5.5 μm and a thickness of 0.25 μm also cannot compete with our results due to the larger width and lower thickness of the meshes.^19^

Our simulations indicate that width and thickness are key parameters in metal mesh transparent EMI shielding performance. However, as described in the Introduction, most metal meshes that have been fabricated thus far have widths greater than 5 μm. Various 3D printing methods such as inkjet, gravure, screen, and flexography are typically limited to line widths of over 10 μm. Creating large thickness metal meshes is also a challenge. Previous transparent EMI shielding work with metal meshes has not been demonstrated with reactive ink-based fabrication approaches. Electroplating and electrodeposition methods are viable options for increasing the thickness of metal meshes, but they are limited because metal tends to deposit isotropically and thus simultaneously increases width. Double-sided structures on both sides of the substrate have demonstrated better performance for transparent EMI shielding,^37^ but these materials require additional steps to fabricate the materials and structures on both sides of the substrate. The demonstrated approach has enabled the highest performance in EMI shielding in the literature for all metal meshes and single-sided EMI shielding materials due to the combination of low conductivity Ag, small widths, and large thicknesses.

## Conclusion

Simulations and experiments on Ag-embedded meshes have been performed for transparent EMI shielding. By using a simple and inexpensive lithography process with Ag ink, we have demonstrated meshes with very high transmission (83.0–90.3% at 550 nm), high EMI shielding efficiency (48.6–58.6 dB), and low sheet resistance (0.46–2.47 Ω per sq). These values represent the best reported performance for transparent EMI shielding for single-sided substrates to date. Further, samples showed little dependency of transmission on wavelength and shielding efficiency on frequency. The use of high conductivity Ag ink and the use of narrow width and large depth trenches in glass enable a combination of high transmission and high SE. Metal meshes with thicknesses as large as 2 μm but only a peak-to-valley difference in height of only about 80 nm with glass are demonstrated. Metal meshes have great potential as transparent EMI shielding for next generation display devices, protective medical devices, and electromagnetic shielding windows.

## Experimental Section

### Ink Information

Commercially available EI-1207, an Ag metal-complex based conductive ink from Electroninks Inc., was used in this study. The ink is based on ink previously developed by one of the authors^38^ and has a viscosity of 8 cps with a solid content of approximately 12%. In the ink, the ammonia ligands compounds act as a stabilizer. When the ink is cured in an oven, the ammonia ligands compounds evaporate and the Ag compound is reduced to form Ag.

### Fabrication of Ag Mesh Glass

The fabrication of glass-embedded Ag meshes is described in Figure 2. Fused silica glass substrates with a dimension of 30 mm × 30 mm were purchased from University Wafers and ultrasonically cleaned in acetone, methanol, and isopropyl alcohol (IPA) for 10 min each, then dried with nitrogen gas. To improve the adhesion of photoresist to the glass, HMDS was sputtered onto the substrate using a vapor prime oven system. The substrate was then spin-coated with a layer of S1805 photoresist for the 1 μm width metal mesh sample with a spin speed of 1500 rpm and with a layer of AZ4110 photoresist for the 2 and 3 μm width metal mesh samples with spin speeds of 2000 and 1000 rpm, respectively. All samples were baked at 110 °C for 5 min. Patterns were made on the photoresist using the Heidelberg MLA100 Direct Write Lithography tool, and were developed for 2 min in AZ400 K (1:4) from MicroChemicals. Reactive ion etching (RIE) was used to transfer the patterns to the glass with a gas flow of 50 sccm Ar and 25 sccm CHF3, a pressure of 30 mT, and a power of 250 W. The depth of the patterns can be controlled by adjusting the etch time, but longer etch times also increase the width of the trenches, leading to lower transmission. Particle-free silver ink (EI-1207 from Electroninks) was spin-coated on the samples at 1000 rpm and then ramp-cured starting at 70 °C with a step increase of 10 °C every 15 min. The final curing temperature is 110 °C. At the final temperature, 30 min was used to completely cure. A razor blade was used to remove the Ag from the glass substrate, leaving Ag only in the trenches. This process was repeated once more to ensure that the trenches were completely filled with Ag. The samples were then spray-washed with acetone, methanol, and IPA to remove any Ag particulates.

### Characterization

A probe station with a semiconductor device analyzer (B1500A Semiconductor Device Analyzer from Keysight Technologies) was used to measure sheet resistance via the van der Pauw method. To get a high resolution images of the glass-embedded Ag meshes, scanning electron microscopy (Zeiss SIGMA VP) was employed. The total transmittance was measured over the wavelength range of 400 to 800 nm using a UV–vis-NIR spectrometer with a 100-mm-diameter integrating sphere (PerkinElmer Lambda 750). The transmission values reported in our study have been calculated by excluding the effect of bare glass transmission. To achieve this, we divided the measured transmission values by the bare glass transmission. The electromagnetic interference shielding effectiveness (EMI SE) was determined using the coaxial transmission line method, with the aid of an HP 7822D Vector Network Analyzer (VNA) for signal generation and detection. The sample was positioned between two waveguide flanges, with the appropriate flange chosen based on the desired frequency range. The waveguide flanges were secured in place using screws and nuts to prevent any shifting during the measurement. The X band and Ku band waveguide flanges were obtained from PASTERNACK.

## Supporting Information Available

Video file 1 (AVI)
Video file 2 (AVI)
Simulations settings, Comparison table of different EMI shielding works in the literature to our work, Reflection and absorption SE contributions, Haze of metal mesh samples, Optical Profilometry, and AFM characterization (PDF)

## Supplementary Material

am3c02088_si_001.avi

am3c02088_si_002.avi
