# Supplementary material for: Silver Meshes for Record-Performance Transparent Electromagnetic Interference Shielding
Source: ACS Appl Mater Interfaces. 2023 Jun 14;15(25):30591–9. doi: 10.1021/acsami.3c02088 (PMC10316324; doi:10.1021/acsami.3c02088)
Supplement: Supplementary file 3 — am3c02088_si_003.pdf [file am3c02088_si_003.pdf]

Supporting Information
Silver Meshes for Record-Performance Transparent Electromagnetic Interference Shielding

Mingxuan Li,[†] Mehdi Zarei,[‡] Khashayar Mohammadi,[¶] S. Brett Walker,[§] Melbs LeMieux,[§] and Paul W Leu*,[‖,‡,†]

†*Department of Chemical Engineering, University of Pittsburgh, Pittsburgh, PA 15261*

‡*Department of Mechanical Engineering, University of Pittsburgh, Pittsburgh, PA 15261*

¶*Department of CivilEngineering, University of Waterloo, Waterloo, N2L3G1, Canada*

§*7901 East Riverside Drive, Bldg 1, Unit 150, Austin, Texas 78744, United States*

‖*Department of Industrial Engineering, University of Pittsburgh, Pittsburgh, PA 15261*

E-mail: pleu@pitt.edu

Table S1: High frequency structure simulator (HFSS) settings for metal mesh EMI simulation

**Parameters**	**Value**
Ag ink conductivity (S/m)	1.3×10^7
Relative permittivity	1
Relative permeability	0.99998
Lande G factor	2
Boundary condition (z-axis)	Perfect E
Boundary condition (x and y-axis)	Periodic
Mesh setting	Default
Frequency sweep (GHz)	8 - 18
Frequency step (GHz)	0.1
Source power (mW)	1
Excitation type	wave port

Table S1 shows the detail settings in the high frequency structure simulator (HFSS) for Ag metal mesh EMI simulation. The metal mesh is modeled as a semi-infinite hexagonal array by using periodic boundary conditions. The Ag is assumed to be 20% that of bulk Ag. The default mesh setting is adaptive, where the software automatically refines the mesh to achieve the desired level of accuracy below the default tolerance of 1%.

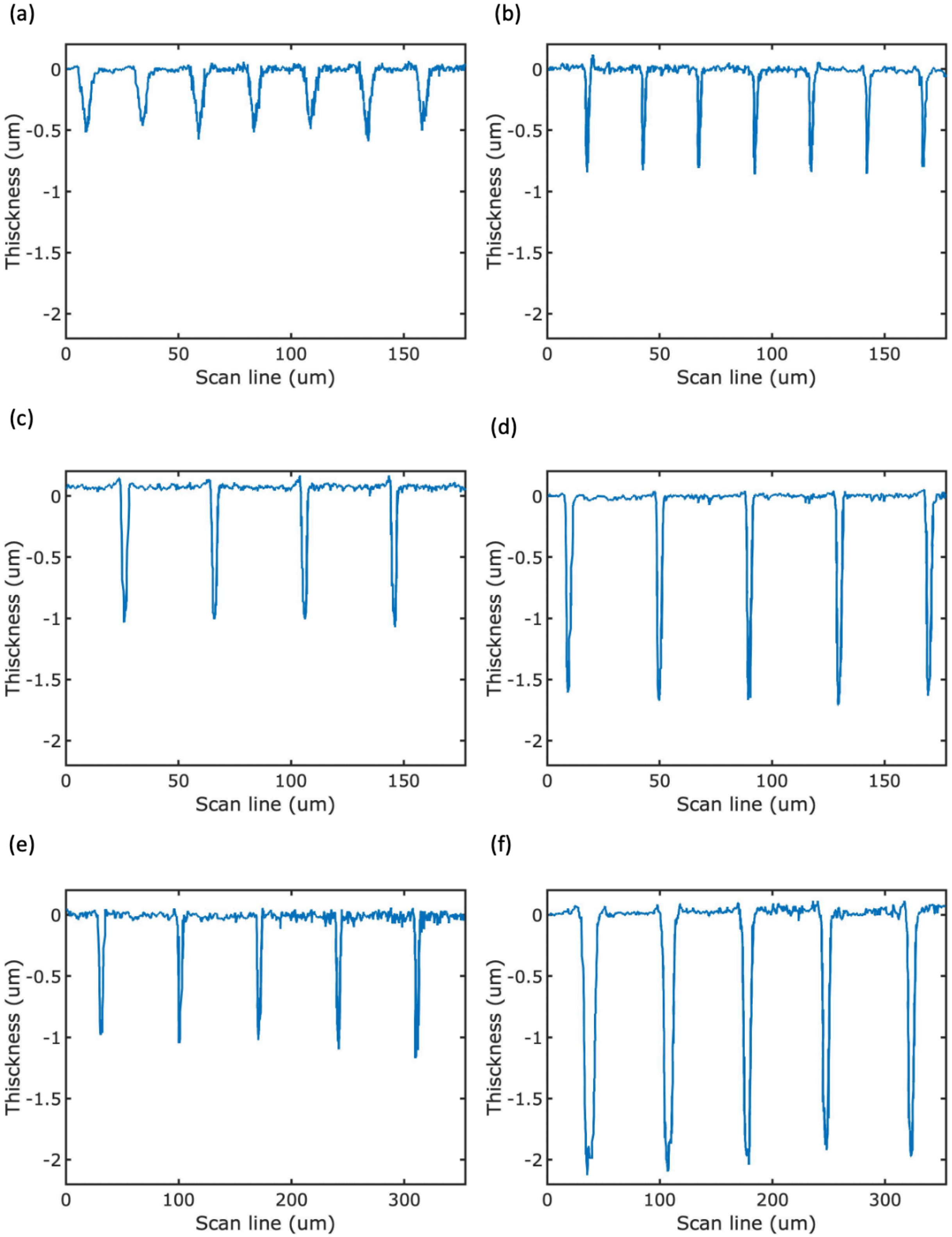

Figure S1: Depth of etched trenches in six studied samples based on optical profilometry measurements for (a) W1P25t0.5, (b) W1P25t0.8, (c) W2P40t1.0, (d) W2P40t1.6, (e) W3P70t1.0, and (f) W3P70t2.0.

Fig. S1 illustrates the depth of the etched trenches in the six studied samples, as determined by optical profilometry measurements. Sample tilt is corrected in the optical profilometry data. The observed variation in trench depth can be attributed to the difference in etch time during the RIE process. The RIE process exhibits a consistent etch rate of 1.2 nm/s, allowing for the reliable fabrication of trenches with uniform depth.

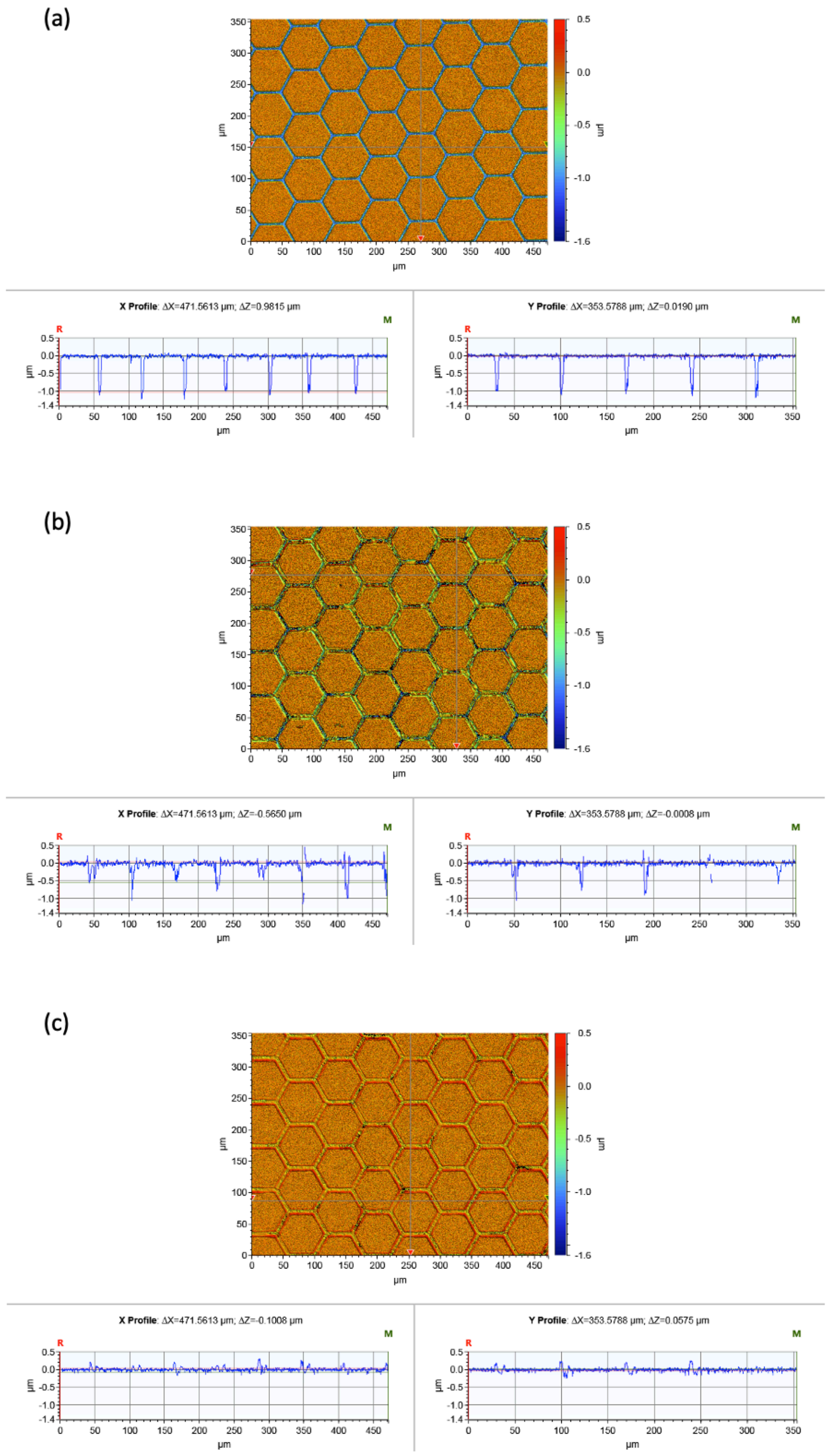

Figure S2: Optical profilometry analysis of W3P70t1.0 sample (a) with zero silver coats, (b) after one pass of silver coat, and (c) after two passes of silver coats.

Fig. S2 shows optical profilometry analysis of the W3P70t1.0 sample (a) prior to silver coating, (b) after one pass of silver coating, and (c) after two passes of silver coating. The left and right scanlines shown under each part correspond to the x and y axes directions, respectively. A uniform thickness of 1 μm was successfully achieved through the RIE process (Fig. S2a). After one silver coat, the trenches are only partially filled to about 0.5 μm due to the large reduction in volume from solvent evaporation during the Ag ink curing process. As a result, a second coat of silver is required to completely fill the trenches. Fig. S2(c) shows that the filled trenches are now uniform with the unetched glass where the scanlines are almost completely flat.

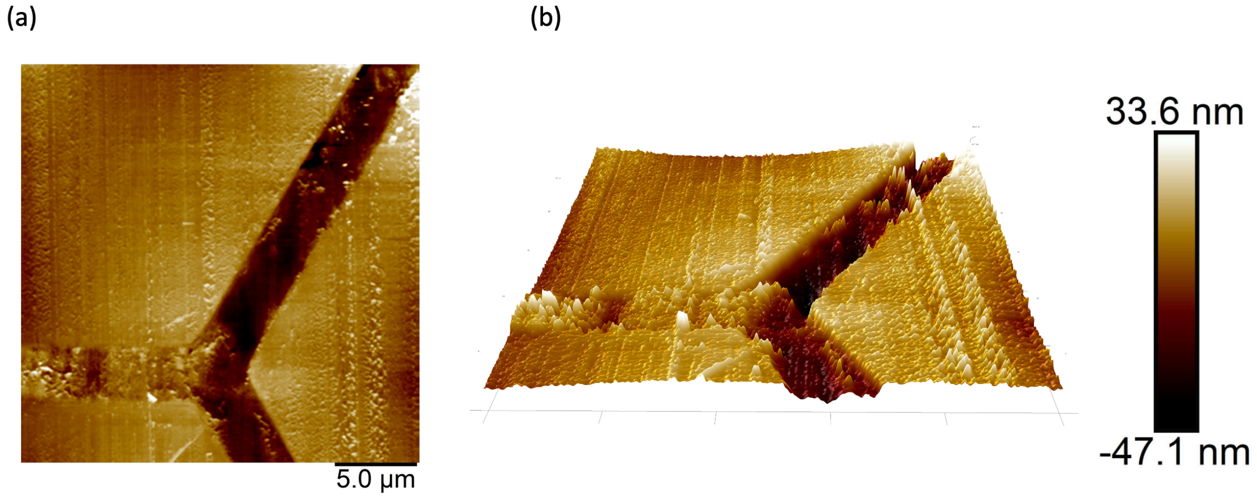

Figure S3: AFM images of W3P70t1.0 sample for (a) 2D and (b) 3D views

Fig. S3 shows 2D and 3D views of atomic force microscopy (AFM) characterization of the W3P70t1.0 sample. The root mean square roughness (R_q) of the silver surface is 6.7 nm, indicating that the top surface is very flat.

Table S2: Haze measurement at 550 nm.

**Sample**	**Total Transmission (%)**	**Scattered Transmission (%)**	**Haze (%)**
W1P25t0.5	87.2	6.1	7.0
W1P25t0.8	83.0	5.1	6.1
W2P40t1.0	87.4	3.8	4.3
W2P40t1.6	83.9	3.7	4.4
W3P70t1.0	90.3	1.1	1.2
W3P70t2.0	84.9	2.0	2.3

Table S2 provides total and scattered transmissions resulting in haze measurement. The transmittance is primarily specular transmittance. However, there is some haze caused by these electrodes, depending on the pitch size. A smaller pitch slightly increases the haze.

Table S3: Comparison of the fabricated samples in this paper with several pioneering works in the literature.

**Reference**	**Material**	**T (%)**	**Frequency (GHz)**	**SE_{ave} (dB)**
W1P25t0.5	Ag mesh	87.2	8-18	48
W1P25t0.8	Ag mesh	83	8-18	58.4
W2P40t1.0	Ag mesh	87.4	8-18	46.3
W2P40t1.6	Ag mesh	83.9	8-18	54
W3P70t1.0	Ag mesh	90.3	8-18	48.3
W3P70t2.0	Ag mesh	84.9	8-18	52.8
Ma *et. al*[1]	Cu/Graphene	91	12-18	25.5
Zhang *et. al*[2]	Graphene/Ag nanowire	78.4	12-18	24
Voronin *et. al*[3]	Ag/Cu mesh	85.4	8-12	38.5
Yuan *et. al*[4]	ZnO/Ag/ZnO mesh	91.9	8-18	34.7
Phan *et. al*[5]	Multi layer salt water	94.2	7.5-8.5	20.5
Wang *et. al*[6]	ITO/Ag-Cu/ITO	96.5	8-18	26
Chen *et. al*[7]	MXene/Ag nanowire	83	8-12	49.2
Walia *et. al*[8]	Cu mesh	85	12-18	41
Jiang *et. al*[?]	Ni mesh	92	8-12	40
Liang *et. al*[9]	Cr/Cu mesh	85	8-18	45
Yang *et. al*[10]	AgNW/rGO networks	91.1	8-12	35
Jiang *et. al*[11]	Ni mesh/ITO glass-double sided	88.7	8-12	42.5

Table S3 shows the comparison of our fabricated samples with other metal meshes in literature. The first six rows show samples from this work. This table provides the material, transmission at 550 nm, frequency range, and average shielding efficiency SE_{ave}. Various frequency ranges are investigated in different papers and SE values are reported as either maximum, minimum, or average. To ensure consistency with this paper, we report the average SE for a frequency range of 8 - 18 GHz.

References

(1) Ma, L.; Lu, Z.; Tan, J.; Liu, J.; Ding, X.; Black, N.; Li, T.; Gallop, J.; Hao, L. Transparent Conducting Graphene Hybrid Films To Improve Electromagnetic Interference (EMI) Shielding Performance of Graphene. *ACS Applied Materials & Interfaces* **2017**, *9*, 34221–34229, PMID: 28892351.

(2) Zhang, N.; Wang, Z.; Song, R.; Wang, Q.; Chen, H.; Zhang, B.; Lv, H.; Wu, Z.; He, D. Flexible and transparent graphene/silver-nanowires composite film for high electromagnetic interference shielding effectiveness. *Science Bulletin* **2019**, *64*, 540–546.

(3) Voronin, A. S.; Fadeev, Y. V.; Govorun, I. V.; Podshivalov, I. V.; Simunin, M. M.; Tambasov, I. A.; Karpova, D. V.; Smolyarova, T. E.; Lukyanenko, A. V.; Karacharov, A. A.; Nemtsev, I. V.; Khartov, S. V. Cu–Ag and Ni–Ag meshes based on cracked template as efficient transparent electromagnetic shielding coating with excellent mechanical performance. *Journal of Materials Science* **2021**, *56*, 14741–14762.

(4) Yuan, C.; Huang, J.; Dong, Y.; Huang, X.; Lu, Y.; Li, J.; Tian, T.; Liu, W.; Song, W. Record-High Transparent Electromagnetic Interference Shielding Achieved by Simultaneous Microwave Fabry–Pérot Interference and Optical Antireflection. *ACS Applied Materials & Interfaces* **2020**, *12*, 26659–26669, PMID: 32422036.

(5) Phan, D. T.; Jung, C. W. Multilayered salt water with high optical transparency for EMI shielding applications. *Scientific Reports* **2020**, *10*, 21549.

(6) Wang, H.; Ji, C.; Zhang, C.; Zhang, Y.; Zhang, Z.; Lu, Z.; Tan, J.; Guo, L. J. Highly Transparent and Broadband Electromagnetic Interference Shielding Based on Ultrathin Doped Ag and Conducting Oxides Hybrid Film Structures. *ACS Applied Materials & Interfaces* **2019**, *11*, 11782–11791.

(7) Chen, W.; Liu, L.-X.; Zhang, H.-B.; Yu, Z.-Z. Flexible, Transparent, and Conductive Ti3C2Tx MXene–Silver Nanowire Films with Smart Acoustic Sensitivity for High-Performance Electromagnetic Interference Shielding. *ACS Nano* **2020**, *14*, 16643–16653, Publisher: American Chemical Society.

(8) Walia, S.; Singh, A. K.; Rao, V. S. G.; Bose, S.; Kulkarni, G. U. Metal mesh-based transparent electrodes as high-performance EMI shields. *Bulletin of Materials Science* **2020**, *43*, 187.

(9) Liang, Z. et al. Metallic nanomesh for high-performance transparent electromagnetic shielding. *Optical Materials Express* **2020**, *10*, 796–806, Publisher: Optical Society of America.

(10) Yang, Y.; Chen, S.; Li, W.; Li, P.; Ma, J.; Li, B.; Zhao, X.; Ju, Z.; Chang, H.; Xiao, L.; Xu, H.; Liu, Y. Reduced Graphene Oxide Conformally Wrapped Silver Nanowire Networks for Flexible Transparent Heating and Electromagnetic Interference Shielding. *ACS Nano* **2020**, *14*, 8754–8765, Publisher: American Chemical Society.

(11) Jiang, Z.; Zhao, S.; Huang, W.; Chen, L.; Liu, Y.-h. Embedded flexible and transparent double-layer nickel-mesh for high shielding efficiency. *Optics Express* **2020**, *28*, 26531–26542, Publisher: Optica Publishing Group.
